# Pre-existing Psychiatric Morbidity Is Strongly Associated to Takotsubo Syndrome: A Case-Control Study

**DOI:** 10.3389/fcvm.2022.925459

**Published:** 2022-07-12

**Authors:** Gino Pozzi, Domenico D'Amario, Giuseppe Princi, Giuseppe Ciliberti, Antonella Irano, Maria Velia Simone, Filippo Crea, Leonarda Galiuto

**Affiliations:** ^1^Catholic University of the Sacred Heart, Rome, Italy; ^2^Department of Aging, Neurological, Orthopedic and Head and Neck Sciences, Fondazione Policlinico Universitario Agostino Gemelli Istituto di Ricovero e Cura a Carattere Scientifico (IRCCS), Rome, Italy; ^3^Department of Cardiovascular Sciences, Fondazione Policlinico Universitario Agostino Gemelli Istituto di Ricovero e Cura a Carattere Scientifico (IRCCS), Rome, Italy

**Keywords:** Takotsubo syndrome, acute coronary syndrome, psychopathology, mood disorders, anxiety disorders

## Abstract

**Background:**

Takotsubo syndrome (TTS) is an emerging disease characterized by an acute and reversible myocardial dysfunction which may have an influence on clinical status and prognosis. Despite extensive research, its pathophysiology has not been completely elucidated; among other hypothesis, a heart-brain interaction has been proposed.

**Methods:**

The aim of this study was to assess the impact of psychiatric disorders and of some personality types on the pathogenesis of TTS. We conducted a retrospective observational case-control study. We enrolled a total of 50 patients, 25 with a previous diagnosis of TTS and 25 patients with a history of acute coronary syndrome (ACS), that underwent a comprehensive lifetime psychiatric assessment.

**Results:**

We found no significant difference between TTS and ACS patients in cardiovascular risk profile. The frequency of lifetime psychiatric disorders was significantly greater in TTS. In particular, in the univariate analysis, TTS group showed a higher prevalence of mood disorders (Major Depressive Disorder, Bipolar Disorder, Dysthymia; 16 vs. 2, *P* < 0.001) and anxiety disorder (Generalized Anxiety Disorder, Panic Disorder, Agoraphobia; 20 vs. 8, *P* = 0.001) compared with ACS group. There was also a significant tendency in TTS patients to psychotropic medication use, substance abuse, and psychologist or psychiatrist consulting. However, there was no difference between the groups in previous stressful events and Type D personality. Moreover, the multivariate analysis showed that mood disorders were independently associated with TTS (OR 16.9, 95% CI, 2.2–127).

**Conclusion:**

Our study demonstrated that pre-existing anxiety disorders and mostly mood disorders were significantly higher in TTS patients than in ACS group, suggesting the role of psychiatric disorders as possible pathophysiological substrate of TTS.

## Introduction

Takotsubo syndrome (TTS) is an emerging cardiovascular disease, characterized by an acute myocardial dysfunction, that predominantly affects postmenopausal women and that is often triggered by emotional or physical stressors (1). It is estimated that 1–3% of all patients and 5–6% of female patients undergoing coronary angiography for suspected acute coronary syndrome (ACS) have TTS (2). TTS usually presents with the occurrence of acute chest pain, dyspnea, palpitations, or syncope. However, it may be diagnosed incidentally by new ischemic electrocardiographic (ECG) changes, ventricular arrhythmias or elevation of cardiac biomarkers. The above findings and the echocardiographic evidence of regional contractile dysfunction make the differentiation between ACS and TTS challenging (2). However, despite the possible coexistence of TTS with ACS, coronary angiography usually does not show obstructive coronary artery disease (CAD) (3), and left ventriculography reveals peculiar and symmetric regional wall motion abnormalities, often in a circumferential apical distribution, hence the name “apical ballooning syndrome” (4).

TTS has long been considered a benign disease, since the ventricular dysfunction recovers within days or weeks (2); however, several studies demonstrated that the mortality of hospitalized patients, during the acute phase, is 4–5%, mainly due to acute heart failure or malignant ventricular arrhythmias, and the rate of long-term complications is high (5, 6). Therefore, a full comprehension of the pathophysiological mechanisms underlying the syndrome and the subsequent use of tailored therapies is crucial to improve outcomes (1).

Nowadays, the pathophysiology of TTS has not been completely clarified and different mechanisms have been proposed (1). Several emotional stressors (e.g., divorce, death of a family member, and financial problems) or physical ones (e.g., physical activity, thyrotoxicosis, sepsis, pregnancy, ACS, and stroke) have been identified as possible triggers of the syndrome (5). These stimuli may promote a sympathetic stimulation, confirmed by increased levels of plasma catecholamines, which may ultimately induce direct toxicity on cardiomyocytes, epicardial, or microvascular coronary artery spasm and acute coronary microvascular dysfunction (7–12). However, only a minority of people who experienced acute stressors then developed TTS, supporting the existence of predisposing substrates on which precipitating factors impact (2).

Based on the role of stressful stimuli and on the evidence of structural anatomical brain differences between TTS patients and healthy controls, a heart-brain interaction has been proposed to play a pivotal role in the development of TTS (13). Furthermore, previous studies have suggested that premorbid psychiatric disease could be an important predisposing risk factor for TTS (14, 15).

The aim of the present study was to assess the influence of psychiatric disorders, as a psychopathological substrate, in patients with TTS compared with patients with ACS.

## Materials and Methods

We designed a retrospective observational case-control study to assess the impact of psychiatric disorders and of some personality types in TTS patients. From December 2018 to December 2021, we enrolled a total of 50 patients, 25 with a previous diagnosis of TTS and 25 patients with a history of ACS. All patients underwent a comprehensive lifetime psychiatric assessment.

The inclusion criterion was a previous diagnosis of TTS (study group) or ACS (control group), while the exclusion criteria included neurodegenerative diseases and ongoing acute psychiatric disorders.

Since all TTS patients were female, we enrolled only women in the control group to exclude the possibility of a gender-related bias.

The diagnosis of TTS was defined according to the “InterTAK criteria” (2): echocardiographic or ventriculographic evidence of apical or midventricular, basal or focal wall motion abnormalities; emotional or physical triggers that precede the onset of symptoms; new ECG ST-T abnormalities; moderately high levels of troponins and brain natriuretic peptides. The control group included patients with previous diagnosis of ST segment Elevation Myocardial Infarction (STEMI), Non-ST segment Elevation Myocardial Infarction (NSTEMI) and Unstable Angina (UA), defined according to the most recent European Guidelines (16, 17).

Both groups of patients were interviewed far from the acute phase (at a minimum of 3-month after discharge) because we assumed that the timing of psychiatric interview may impact on reliability and reproducibility of the diagnosis. In particular, the distress related to hospitalization for acute cardiovascular disease may hinder or exalt the accurate diagnosis of psychiatric disorders.

We acquired in-hospital clinical-instrumental data, including demographics assessment, cardiovascular risk profile, pharmacological anamnesis, echocardiographic evaluation, and angiographic data. All patients were questioned about their medical history and lifestyle, including the use of psychotropic drugs or substance abuse.

Several instruments were used to assess the psychological and psychopathological profile of TTS and ACS patients, including: the Emotion Regulation Questionnaire (ERQ), performed to evaluate individual tendencies to reappraise and to suppress the expression of emotions (18); the Connor-Davidson Resilience Scale (CD-RISC), used to measure stress coping ability and stress reactions (19); the DS-14, a measure of negative affectivity and social inhibition, typical features of Type D personality (20); the PID-5, a useful screening tool for personality disorders (21); the Mini International Neuropsychiatric Interview (MINI), a short structured diagnostic interview administered to diagnose the most frequent mental disorders (22) in a revised Italian version according to the current Diagnostic and Statistical Manual of Mental Disorders (DSM-5) criteria; the Social Readjustment Rating Scale (SRRS), a questionnaire to identify major stressful life events (23).

Statistical analysis was performed using statistical software package Statistic for Data Analysis (SPSS) version 27. In the univariate analysis, Chi-square or Fisher's exact tests were used to compare categorical variables between TTS patients and ACS patients. Continuous variables were listed as mean ± standard deviation (SD) and were compared between groups using *t*-tests. A two-side *P*-value < 0.05 was required for statistical significance. Moreover, multivariable regression analysis was performed and Adjusted Odd Ratios (OR) with 95% confidence intervals (95% CI) were calculated.

## Results

Clinical characteristics of the two groups are presented in Table 1: TTS and ACS patients are all female, and the mean age is respectively 67 ± 11.4 years old and 71.2 ± 10.3 years old in the two groups. We found no significant difference in TTS and ACS patients in cardiovascular risk profile (dyslipidemia, diabetes, hypertension, smoke, body mass index, cardiovascular family history, chronic kidney disease and hyperuricemia). Furthermore, no significant differences were found in previous episodes of TTS or ACS. The frequency of psychiatric disorders was significantly greater in patients with TTS compared with ACS patients. In particular the TTS group showed a higher prevalence of mood disorders (MD), i.e., Major Depressive Disorder, Bipolar Disorder, and Dysthymia (16 vs. 2, *P* < 0.001), anxiety disorders (AD), i.e., Generalized Anxiety Disorder, Panic Disorder and Agoraphobia (20 vs. 8, *P* = 0.001), and other disorders, i.e., Post-Traumatic Stress Disorder, Eating disorders and Alcohol Use Disorder (8 vs. 2, *P* = 0.034) compared with the control group (Table 2). Moreover, among patients with TTS, more than half of them received multiple diagnosis of psychiatric disorders in their lifetime.

**Table 1:** Clinical features of TTS and ACS patients.

	**TTS (*n* = 25)**	**ACS (*n* = 25)**	***P*-value**
Age (mean ± SD)	67 ± 11.4	71.2 ± 10.3	0.169
Female sex	25 (100%)	25 (100%)	1
Diabetes	2 (8%)	3 (12%)	0.751
Hypertension	10 (40%)	15 (60%)	0.316
Dyslipidemia	11 (44%)	15 (60%)	0.604
Smoker	12 (48%)	8 (32%)	0.087
BMI	23.1 ± 3.5	24.2 ± 3.9	0.19
CV family history	8 (32%)	10 (40%)	0.895
CKD	2 (8%)	2 (8%)	0.855
Hyperuricemia	1 (4%)	3 (12%)	0.363
Polivascular disease	1 (4%)	2 (8%)	0.658
Previous ACS	1 (4%)	2 (8%)	0.658
Previous TTS	1 (4%)	0	0.270

*Values are n (%) or mean ± SD*.

*TTS, Takotsubo Syndrome; ACS, Acute Coronary Syndrome*.

**Table 2:** Psychiatric features of TTS and ACS patients.

	**TTS (*n* = 25)**	**ACS (*n* = 25)**	***P*-value**
Mood disorders	16 (64%)	2 (8%)	<0.001
Anxiety disorders	20 (80%)	8 (32%)	0.001
Other disorders	8 (32%)	2 (8 %)	0.034
Previous stressful events	25 (100%)	22 (88%)	0.07
D personality	4 (16%)	2 (8%)	0.38
Psychiatrist/psychologist	16 (64%)	3 (12%)	0.01
Psychotropic drugs	18 (72%)	7 (28%)	0.02
Substance abuse	5 (20%)	0	0.018
Cognitive restructuring	28.5 ± 8.7	28.4 ± 9.86	0.9
Response suppression	13.3 ± 5.8	14.7 ± 6.4	0.4
Negative affectivity	12.2 ± 5.9	14.8 ± 5.4	0.1
Social inhibition	7 ± 4.3	5.8 ± 3.1	0.28
CD-RISK	64.7 ± 13.8	65.36 ± 15.2	0.88
Language/learning problems	1 (4%)	2 (8%)	0.5

*Values are n (%) or mean ± SD*.

*TTS, Takotsubo Syndrome; ACS, Acute Coronary Syndrome; CD-RISK, Connor Davidson Resilience Scale*.

In confirmation of the above findings, there was a relevant tendency for TTS patients to psychologist or psychiatrist consulting (16 vs. 3, *P* = 0.01) but also to make use of psychotropic medication (18 vs. 7, *P* = 0.02) and substances of abuse (5 vs. 0, *P* = 0.018) (Table 2). Conversely, there was no difference between the groups in previous stressful events (25 vs. 22, *P* = 0.07) and Type D personality (negative affectivity and social inhibition; 4 vs. 2, *P* = 0.38; Table 2). In order to increase the strength of these associations and to evaluate the influence of confounding factors on final results, we created a multivariate logistic regression model adjusted for the six variables whose differences were statistically significant on univariate analysis between the two groups (mood disorders, anxiety disorders, other psychiatric disorders, psychiatrist/psychologist, psychotropic drugs, and substance abuse). Mood disorders were independently associated with TTS, OR 16.9 (95% CI 2.2–127).

## Discussion

Since its first description in 1990, TTS has gained worldwide recognition (5). Even if several mechanisms have been proposed, such as catecholamine-induced myocardial toxicity (8), epicardial or microvascular coronary artery spasm (9, 10) and acute coronary microvascular dysfunction, the etiopathogenesis of TTS is still matter of debate (1). We hypothesized that the susceptibility to TTS could be related to a history of pre-morbid psychiatric disorders. To test this assumption, we performed a retrospective case-control study in which 25 patients with previous diagnosis of TTS and as many with previous diagnosis of ACS underwent a comprehensive lifetime psychiatric assessment, using a systematic interview.

In our study, among TTS patients, the diagnosis of either MD (16 vs. 2, *P* < 0.001) or AD (20 vs. 8, *P* = 0.001) were significantly higher than in ACS group. In particular, MD was independently associated with TTS, OR 16.9 (95% CI 2.2–127). MD and AD were found, respectively, in 64% and 80% of TTS patients, much more than the estimated lifetime prevalence in the general population (24). Moreover, more than half of TTS patients (14; 56%) received multiple diagnosis of psychiatric disorders.

Other studies have yielded similar results: in particular, a case-control study showed an association between TTS with depressive disorders, but not with anxiety syndromes (25) and other authors demonstrated a higher frequency of depressive and anxiety disorders in TTS patients compared to ACS patients (14, 15); contrarily two case-control studies showed that higher levels of anxiety, but not depression, were associated with the occurrence of TTS (26, 27).

Moreover, in our study the prevalence of “other disorders” (Post-Traumatic Stress Disorder, Eating disorders and Alcohol Use Disorder) was significantly higher (8 vs. 2, *P* = 0.034) in patients with TTS compared to control group.

Nowadays, it is well-known that TTS is predominantly preceded by emotional or physical stressors (5). All TTS patients have experienced at least one stressful event; however, it was not significantly higher compared to ACS group (25 vs. 22, *P* = 0.07). Probably, this result might support the existence of predisposing substrates that underlie the development of TTS. Moreover, despite proposed as a key role for increasing biological reactivity to acute emotional stress, the prevalence of Type D personality (characterized by negative affectivity and social inhibition) (28) did not significantly differ between TTS and ACS patients (4 vs. 2, *P* = 0.38).

TTS patients showed a high prevalence of cardiovascular risk factors, similar to ACS group. Probably, the progression of cardiovascular risk factors might have promoted the development of endothelial dysfunction (29), a common pathophysiological feature of TTS (30). In this regard Naegele et al. demonstrated that endothelial dysfunction is common in patients with TTS, which also suggests the propensity for coronary artery spasm (31).

Finally, in confirmation of the importance of psychiatric disorders, TTS patients made use of psychotropic medication (18 vs. 7, *P* = 0.02) and substances of abuse (5 vs. 0, *P* = 0.018), and consulted psychologist or psychiatrist (16 vs. 3, *P* = 0.01) more frequently than ACS patients.

Our findings might support the role of psychiatric disorders as possible correlate of neurologically mediated susceptibility to TTS, reinforcing the concept of the heart-brain interaction (32).

In this regard, some studies documented alterations of brain regions involved in autonomic functions and in regulation of the limbic system (13, 33). In particular, Templin et al. suggested that patients with TTS have structural brain alterations and hypoconnectivity of the amygdala, a component of the limbic system that notably plays a pivotal role in the response to acute stressors (13). Moreover, evidences showed that amygdalar activity independently predicts cardiovascular events (34). Interestingly, Radfar et al., through 18F-FDG-PET/CT imaging, demonstrated that baseline amygdalar activity is higher in individuals who subsequently develop TTS, supporting the existence of a predisposing neurobiological substrate of TTS (35).

**Figure 1:**
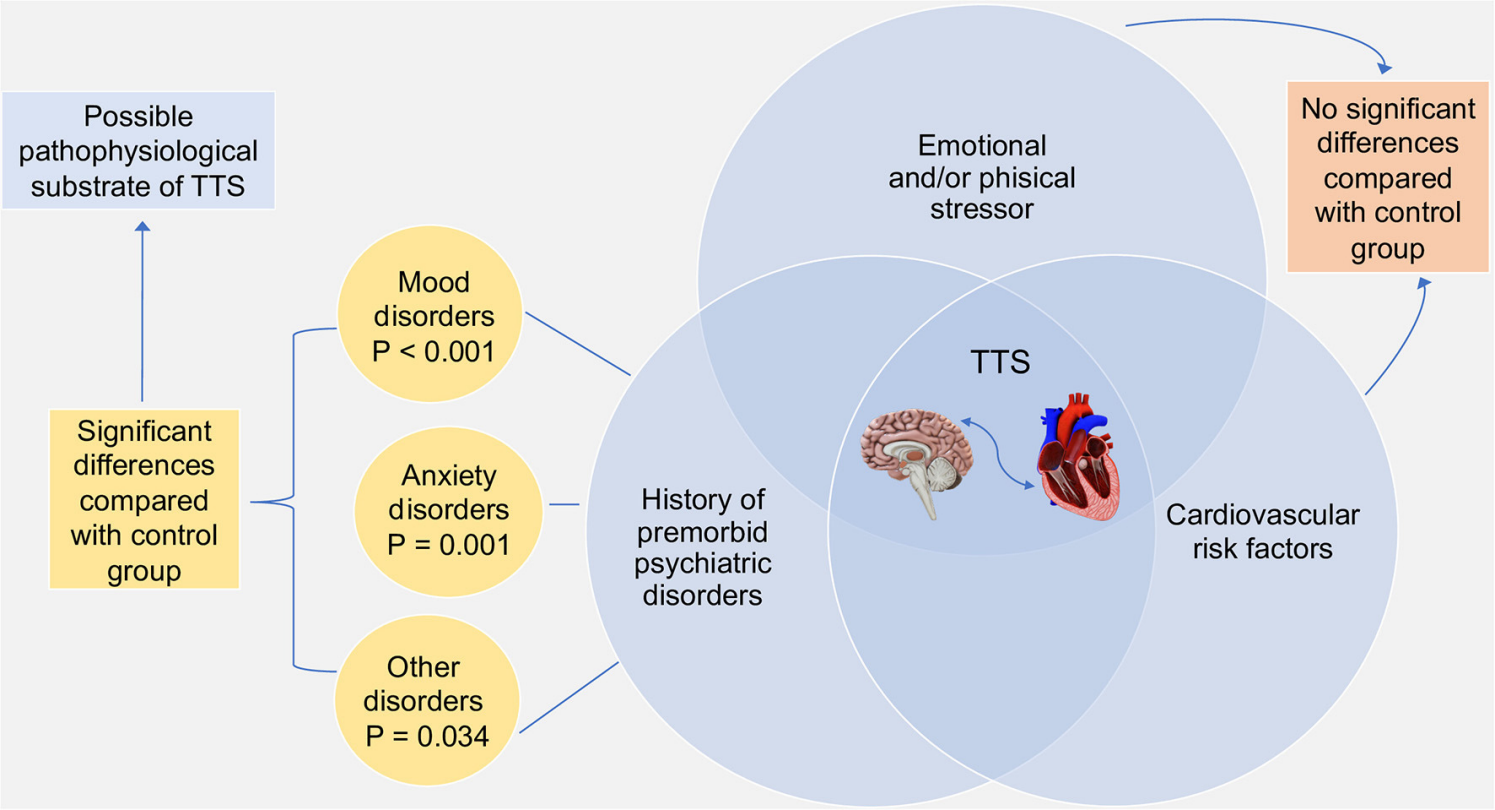
Pathophysiological substrates of TTS and major results of the study.

Our study demonstrated that pre-existing anxiety disorders and mostly mood disorders were significantly higher in TTS patients than in ACS group, suggesting the role of psychiatric disorders as possible pathophysiological substrate of TTS, using, for the first time, a lifetime systematic psychiatric assessment performed far from the acute phase (Figure 1).

This study has potential limitations. Firstly, there is a small-size population, which may make the result inaccurate because the data is not enough: only 50 participants. Secondly, the retrospective nature of the study gives an inferior level of evidence and the lack of follow-up data does not allow to determine whether psychiatric morbidity also have a prognostic role. Further studies are needed to confirm our findings in larger cohorts and to determine the role of MD and AD in the pathophysiology of TTS.

## Data Availability Statement

The original contributions presented in the study are included in the article/supplementary material, further inquiries can be directed to the corresponding author.

## Ethics Statement

The studies involving human participants were reviewed and approved by Ethics Committee Fondazione Policlinico Universitario Agostino Gemelli Rome. The patients/participants provided their written informed consent to participate in this study.

## Author Contributions

LG, FC, DD'A, and GPo conducted the study. GPr, GC, AI, and MS supported the realization of the study. GPr and GC had a leading role in writing the manuscript. LG, FC, DD'A, and GPo had a leading role in manuscript revision. All authors have read and agreed to the content of the manuscript.

## Conflict of Interest

The authors declare that the research was conducted in the absence of any commercial or financial relationships that could be construed as a potential conflict of interest.

## Publisher's Note

All claims expressed in this article are solely those of the authors and do not necessarily represent those of their affiliated organizations, or those of the publisher, the editors and the reviewers. Any product that may be evaluated in this article, or claim that may be made by its manufacturer, is not guaranteed or endorsed by the publisher.
